# Anti-VEGF antibody triggers the effect of anti-PD-L1 antibody in PD-L1^low^ and immune desert-like mouse tumors

**DOI:** 10.3892/or.2021.8247

**Published:** 2021-12-23

**Authors:** Nobuyuki Ishikura, Masamichi Sugimoto, Keigo Yorozu, Mitsue Kurasawa, Osamu Kondoh

**Affiliations:** Product Research Department, Chugai Pharmaceutical Co., Ltd., Kamakura, Kanagawa 247-8530, Japan

**Keywords:** anti-programmed death-ligand 1, anti-vascular endothelial growth factor, atezolizumab, bevacizumab, C-X-C motif chemokine receptor 3 ligands, immune desert

## Abstract

The efficacy of programmed cell death-ligand 1 (PD-L1)/programmed cell death protein 1 (PD-1) blockade therapy has been demonstrated but is limited in patients with PD-L1^low^ or immune desert tumors. This limitation can be overcome by combination therapies that include anti-vascular endothelial growth factor (VEGF) therapy. Such combinations have been investigated in clinical trials for a number of cancer types; however, evidence on the mechanisms underlying their effects in these types of patients is still not sufficient. Therefore, the present study investigated the efficacy and effects on CD8^+^ T cell and C-X-C motif chemokine receptor 3 (CXCR3) ligand expression in tumors by combining anti-PD-L1 and anti-VEGF antibodies using an OV2944-HM-1 mouse model with PD-L1^low^ and immune desert-like phenotypes. Although the model exhibited anti-PD-L1 insensitivity, anti-PD-L1 antibody treatment combined with anti-VEGF antibody inhibited tumor growth compared with anti-VEGF monotherapy, which itself inhibited tumor growth compared with the control treatment on Day 25. In combination-treated mice, a higher percentage of CD8^+^ T cells and higher levels of CXCR3 ligands were observed in tumor tissues compared with those in the anti-VEGF antibody treatment group, which was not significantly different from control treatment on Day 8. The increase in the intratumoral percentage of CD8^+^ T cells following the combination treatment was reversed by CXCR3 blocking to the same level as the control. In an anti-PD-L1 insensitive model with PD-L1^low^ and immune desert-like phenotypes, although anti-PD-L1 antibody alone was not effective, anti-PD-L1 antibody in combination with anti-VEGF antibody exhibited antitumor combination efficacy with an increase of CD8^+^ T cell infiltration, which was suggested to be dependent on the increase of intratumoral CXCR3 ligands. This mechanism could explain the efficacy of anti-PD-L1 antibody and anti-VEGF antibody combination therapy in the clinical setting.

## Introduction

Programmed cell death-ligand 1 (PD-L1) is an immune checkpoint molecule expressed on tumor cells and tumor-infiltrating immune cells, which is involved in the suppression of cancer immunity (1). Anti-PD-L1 antibody relieves T cell suppression by inhibiting the binding of PD-L1 to programmed cell death protein 1 (PD-1) and B7.1 (also known as CD80), which are receptors on effector T cells, and exerts antitumor effects in various types of cancer (2). In a phase 3 OAK trial, atezolizumab (anti-PD-L1 antibody) treatment prolonged overall survival compared with docetaxel in previously treated patients with non-small cell lung cancer, regardless of PD-L1 expression status (3). However, intratumor PD-L1 expression is generally considered to enrich patients for whom anti-PD-L1/PD-1 therapy would most likely be efficacious, and tumors with the immune-desert phenotype (low CD8-positive rate) also rarely respond to anti-PD-L1/PD-1 therapy as a single agent (4). To expand the benefit of these antibodies, numerous combination strategies, e.g. with bevacizumab, chemotherapy and ipilimumab, have been extensively investigated (5–8).

Vascular endothelial growth factor (VEGF) has been reported to exert not only tumor angiogenesis-inducing activity, but also immunosuppressive activity which can attenuate the antitumor immunity elicited by anti-PD-L1/PD-1 therapy through inhibition of dendritic cell (DC) maturation (9–12) and accumulation of myeloid-derived suppressor cells (MDSCs) (13). It has been reported that VEGF blockade may promote antitumor immunity by inhibiting the accumulation of regulatory T-cells (Tregs) (14).

Therefore, the combination of anti-PD-L1/PD-1 antibody and anti-VEGF antibody has been actively investigated in clinical studies of numerous types of cancer, such as non-small cell lung cancer, hepatocellular carcinoma, ovarian cancer and renal cell carcinoma (5,15,16). The IMpower150 clinical trial conducted on non-squamous non-small cell lung cancer demonstrated that the combination of atezolizumab plus bevacizumab (anti-VEGF antibody) and chemotherapy markedly prolonged the progression-free and overall survival of patients with metastatic non-squamous non-small cell lung cancer (5). Although several possible mechanisms for the combination of PD-L1/PD-1 and VEGF blockades have been reported using anti-PD-L1/PD-1 blockade-sensitive models (17–19), to the best of our knowledge, no studies have used a PD-L1^low^ and immune desert-like tumor model.

The present study investigated the efficacy and mechanisms of an anti-PD-L1 and anti-VEGF combination in an anti-PD-L1 insensitive OV2944-HM-1 (HM-1) mouse model with PD-L1^low^ and immune desert-like phenotypes.

## Materials and methods

### Cell lines and culture conditions

OV2944-HM-1 (HM-1) murine ovarian cancer cells were purchased from RIKEN BioResource Center and maintained in MEM Alpha (Thermo Fisher Scientific, Inc.) supplemented with 10% FBS (Bovogen Biologicals Pty Ltd.) (20). Colon 38 murine colon cancer cells were obtained from the Japanese Foundation for Cancer Research based on a Material Transfer Agreement with the National Cancer Institute and were maintained in RPMI-1640 (Merck KGaA) supplemented with 10% FBS. Both cell lines were incubated with 5% CO_2_ at 37°C.

### Animals

A total of 708 female 6–8-week-old B6C3F1 mice were purchased from CLEA Japan, Inc. for the HM-1 model. A total of 80 female 7-week-old C57BL/6J mice were purchased from Charles River Laboratories, Inc. for the Colon 38 model. All animals were housed in a specific pathogen-free environment under controlled conditions (temperature, 20–26°C; humidity, 35–75%; 12 h light/12 h dark cycle), and were allowed to acclimate and recover from shipping-related stress for 5 days or more prior to the study. Chlorinated water and irradiated food were provided *ad libitum.* The health of the mice was monitored by daily observation. Mice at the time of tumor inoculation and at the time of randomization were 6–11 weeks old and 8–12 weeks old, respectively. The body weights of the B6C3F1 mice and C57BL/6J mice at the time of randomization were 19.2-25.8 and 18.7-21.7 g, respectively. After the experiments, all animals from which tumor tissues were not obtained were euthanized by CO_2_ asphyxiation with a CO_2_ displacement rate of 20% of the chamber volume per min, followed by cervical dislocation; and the animals from which tumor tissues were obtained were euthanized by exsanguination under 2.0-2.5% isoflurane inhalation anesthesia using isoflurane inhalation solution (Pfizer, Inc.). Animal death was confirmed by the loss of signs, such as response to toe pinch and heartbeat. Finally, graying of the mucous membranes and rigor mortis were confirmed. All animal experiments were reviewed and approved by the Institutional Animal Care and Use Committee at Chugai Pharmaceutical Co., Ltd. (approval nos. 15-114 and 17-059) and were conducted between February 2017 and February 2019.

### In vivo tumor growth inhibition studies

HM-1 tumor cells (1×10^6^ cells) in 100 µl MEM Alpha (Thermo Fisher Scientific, Inc.) were subcutaneously inoculated into the right flank of B6C3F1 mice. Colon 38 tumor cells (5×10^6^ cells) in 100 µl 50% Matrigel Growth Factor Reduced Basement Membrane Matrix (Corning, Inc.)-RPMI-1640 (Merck KGaA) were subcutaneously inoculated into the right flank of C57BL/6J mice. Mice with established tumors were randomly allocated to each treatment group (Day 1). The time intervals between tumor inoculation and randomization were 9–16 and 14 days in HM-1 and Colon 38 models, respectively. For treatment, anti-mouse PD-L1 monoclonal antibody (mAb; clone 6E11; provided by Genentech, Inc., not commercially available), which blocks the binding of both PD-L1 to PD-1 and PD-L1 to B7-1 (CD80) (21), and anti-mouse VEGF mAb (clone B20-4.1.1; provided by Genentech, Inc., not commercially available), were used. Optimized for recombinant production in mammalian cells (22), B20-4.1.1 is a variant of B20-4.1, an antibody that prevents both human VEGF and mouse VEGF from binding VEGFR2 and VEGFR1 with high potency (23). Anti-mouse PD-L1 mAb or mouse IgG (SouthernBiotech) was administered intraperitoneally to the mice at a dose of 5 mg/kg twice a week from Day 1. Anti-mouse VEGF mAb or mouse IgG was administered intraperitoneally to the mice at a dose of 10 mg/kg weekly from Day 1. For CD8 depletion, anti-mouse CD8 mAb (clone 116-13.1; cat. no. BE0118; Bio X Cell) or Rat IgG (cat. no. 55951; MP Biomedicals) was administered intraperitoneally to the mice at a dose of 100 µg/mouse twice a week from 11 days before randomization. For C-X-C motif chemokine receptor 3 (CXCR3) blocking, anti-mouse CXCR3 mAb (clone CXCR3-173; cat. no. 126538; BioLegend, Inc.) or hamster IgG (cat. no. 402020; BioLegend, Inc.) was administered intraperitoneally to the mice at a dose of 100 µg/mouse twice a week from Day 1. The time intervals between tumor inoculation and final tumor growth measurement were 14–35 and 36 days in HM-1 and Colon 38 models, respectively.

Tumor volume was measured twice a week. Tumor volume was estimated using the following equation: Tumor volume = ab^2^/2, where a and b are the tumor length and width (a≥b), respectively.

### Flow cytometry analysis

For the analysis of tumor-infiltrating lymphocytes, tumor tissue was excised from control-treated mice and antitumor agent-treated mice, and single-cell suspensions were obtained by mincing tumors and homogenizing them by disruption and digestion with a gentleMACS Octo Dissociator with Heaters (Wakenyaku Co., Ltd.) and a Tumor Dissociation Kit for mice (Miltenyi Biotec GmbH). Single-cell suspensions were incubated with anti-mouse CD16/CD32 (Fcγ receptor) antibodies (2.4G2; Tonbo Biosciences, cat. no. 70-0161) and the fixable viability dye (FVD) eFluor 506 or FVD780 (Thermo Fisher Scientific, Inc., cat. no. 65-0866-14 or 65-0865-14, respectively) at 4°C for 5 min, and stained with the following anti-mouse monoclonal antibodies: PerCP-Cy5.5 anti-CD45 [30-F11; cat. no. 550994; used for assays that did not involve anti-granzyme B (Gzm) and anti-interferon (IFN)-γ], BV650 anti-CD8α (53–6.7; cat. no. 563234; used for assays that did not involve anti-GzmB and anti-IFN-γ), PE anti-CD8α (53–6.7; cat. no. 553033; used for assays involving anti-GzmB and anti-IFN-γ), Alexa Fluor 674 anti-GzmB (GB11; cat. no. 560212), BV711 anti-CD11c (HL3; cat. no. 563048), PE-CF594 anti-F4/80 (T45-2342; cat. no. 565613), BV650 anti-CD4 (RM4-5; cat. no. 563747; used for assays that did not involve anti-CXCR3), PE-Cy7 anti-CD4 (RM4-5; cat. no. 552775; used for assays involving anti-CXCR3), BV510 anti-CD11b (M1/70; cat. no. 562950), PE-Cy7 anti-FasL (CD95; Jo2; cat. no. 557653), APC anti-intercellular adhesion molecule-1 (ICAM-1, CD54; 3E2; cat. no. 561605) and BV605 anti-vascular cell adhesion molecule 1 (VCAM-1, CD106; 429; cat. no. 745193) from BD Biosciences; FITC anti-major histocompatibility complex (MHC) class I (H-2K^k^; 36-7-5; cat. no. 114905), PE-Cy7 anti-CD80 (16–10A1; cat. no. 104734), BV605 anti-CD86 (GL1; cat. no. 105037) and PE anti-CD31 (390; cat. no. 102408) from BioLegend, Inc.; PE-Cy5.5 anti-forkhead box P3 (Foxp3; FJK-16s; cat. no. 35-5773-82) and Alexa Fluor 700 anti-granulocyte-differentiation antigen (Ly-6G/Ly-6C (Gr-1); RB6-8C5; cat. no. 56-5931-80) from Thermo Fisher Scientific, Inc.; and FITC anti-CD45 (30-F11; cat. no. 35-0451; used for assays involving anti-Gzm B and anti-IFN-γ) and APC anti-IFN-γ (XMG1.2; cat. no. 20-7311) from Tonbo Biosciences. Appropriate conjugated isotype-matched IgG was used as the control for each. Intracellular cytokines were stained using a Foxp3/Transcription Factor Staining Buffer Set (Thermo Fisher Scientific, Inc.). Cells were analyzed using an LSRFortessa X-20 cell analyzer (BD Biosciences) and FlowJo 10 software (Tree Star, Inc.).

### Immunohistochemistry

PD-L1 expression at baseline in tumor tissues was evaluated by immunohistochemical staining using anti PD-L1 antibody (goat anti-mouse B7-H1/PD-L1 polyclonal antibody; dilution, 1:4,000; cat. no. AF1019; R&D Systems, Inc.) as a primary antibody. CD8α^+^ T cells in tumor tissues were evaluated at baseline and on Day 8 by immunohistochemical staining using anti-CD8 antibody [rat anti-mouse CD8 alpha monoclonal antibody KT15; dilution, 1:500 (baseline) or 1:800 (Day 8); cat. no. GTX76351; GeneTex, Inc.] as a primary antibody. Tumor samples were collected on Day 1 without the drug treatment or on Day 8 with the drug treatments. Fresh frozen blocks were prepared from the collected tumors with optimal cutting temperature compound (O.C.T. compound) at −78°C. Subsequently, 5-µm-thick sections from fresh frozen tissues were fixed in 4% paraformaldehyde at 4°C for 10 min. The endogenous peroxidase activity and endogenous non-specific background were blocked with 0.3% hydrogen peroxide in methanol at room temperature for 30 min. The tissue sections were incubated at 4°C overnight with anti PD-L1 antibody or anti CD8 antibody as the primary antibody. Subsequently, the sections were incubated at room temperature with the Universal Immuno-peroxidase Polymer reagent (undiluted; N-Histofine^®^ Simple Stain Mouse MAX-PO (G); cat. no. 414351; Nichirei Bioscience, Inc.) for 15 min or the Universal Immuno-peroxidase Polymer reagent (undiluted; N-Histofine^®^ Simple Stain Mouse MAX-PO(Rat); cat. no. 414311; Nichirei Bioscience, Inc.) for 30 min, respectively. Staining was conducted at room temperature using 3,3-diaminobenzidine solution (DAB+, Liquid, 2-component system; cat. no. K3468; Agilent Technologies, Inc.) for 5 min. All sections were counterstained at room temperature with hematoxylin for 1–3 sec. Histological examination was performed under a light microscope (Nikon ECLIPSE Ni; Nikon Corporation) in a blinded manner. The evaluation was performed by an experienced pathologist. Immunohistochemical scoring of CD8α^+^ T cells was carried out using grades of 0-3: 0, none; 1, scattered cell infiltration with 0 or 1 focal cell infiltration in a specimen; 2, scattered cell infiltration with 2–4 focal cell infiltrations in a specimen; and 3, diffuse cell infiltration or ≥5 focal cell infiltrations in a specimen.

### Mouse C-X-C motif chemokine ligand (CXCL)9, CXCL10, CXCL11 and IFN-γ ELISA assay

Tumor tissues collected from mice and stored at −80°C were homogenized with Cell Lysis buffer (Cell Signaling Technology, Inc.) with Complete Protease Inhibitor Cocktail Tablets and Complete Phosphatase Inhibitor Cocktail Tablets (both from Roche Diagnostics). The homogenate was centrifuged at 9,100 × g at 4°C for 20 min. The resultant supernatant was used for the assays as cell lysate. Protein concentration of the cell lysates was quantified using a Direct Detect spectrometer (Merck KGaA). The following manufacturers' kits were used for Mouse CXCL9/MIG Quantikine ELISA Kit (cat. no. MCX900; R&D Systems, Inc.), Mouse IP-10 (CXCL10) ELISA Kit (cat. no. BMS6018; Thermo Fisher Scientific, Inc.), Mouse C-X-C motif chemokine 11 (CXCL11) ELISA kit (cat. no. CSB-EL006241MO; Cusabio Technology LLC) and Mouse IFN-γ Quantikine ELISA Kit (cat. no. MIF00; R&D Systems, Inc.).

### Immunohistochemistry and quantification of microvessel density (MVD) in tumor tissues

MVD in tumor tissues was evaluated by immunohistochemical staining of CD31. Tumor samples were collected on Day 8. Fresh frozen blocks were prepared from the collected tumors with O.C.T. compound at −78°C. Immunohistochemical staining was conducted as described previously (24). In brief, immunohistochemical analysis of CD31 was conducted using a Rat HRP-Polymer 1-Step (mouse adsorbed) system (cat. no. BRR4016; Biocare Medical, LLC) according to the manufacturer's protocols. As the primary antibody, rat anti-mouse CD31 monoclonal antibody (clone MEC 13.3; dilution, 1:500; cat. no. 553370) was purchased from BD Biosciences. MVD (%) was calculated from the ratio of the CD31-positive staining area to the total observation area in the viable region. Positive staining areas were calculated using imaging analysis software (Definiens Tissue Studio; version 3.60; Definiens, Inc.).

### Statistical analysis

The experiments were conducted twice and data from the two experiments, which showed a similar trend, were pooled and presented as the mean ± standard deviation. For comparisons between two groups, data were analyzed using the Wilcoxon rank sum test. P<0.05 was considered to indicate a statistically significant difference. For multiple comparisons, data were analyzed with the Wilcoxon rank sum test, and then the P-values were corrected using the Holm-Bonferroni method. Corrected P-values <0.05 were considered to indicate a statistically significant difference (25). All statistical analyses were conducted using JMP software (version 15; SAS Institute, Inc.).

## Results

### HM-1 model exhibits PD-L1^low^ and immune desert-like phenotypes

**Figure 1.**
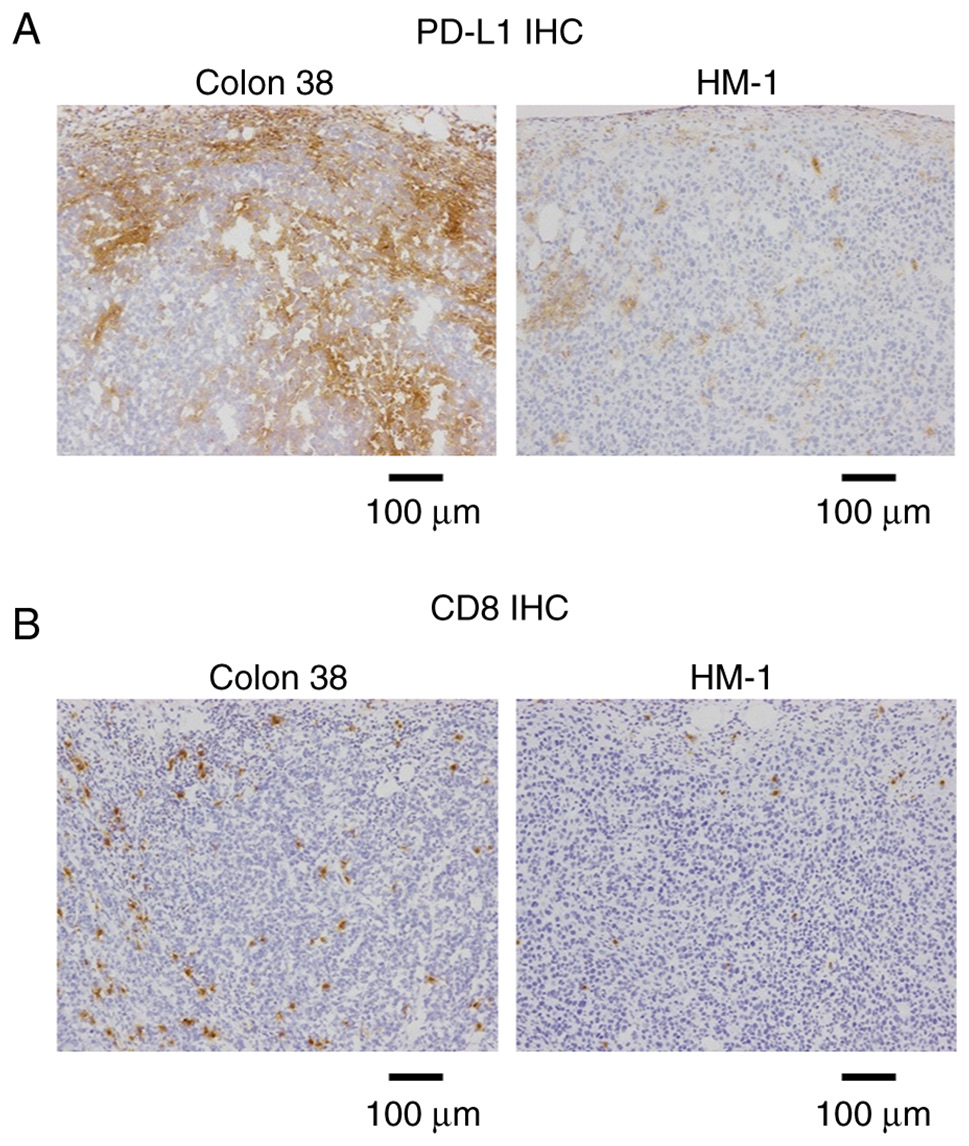
PD-L1 expression and CD8^+^ infiltration at baseline. (A) PD-L1 immunostaining at baseline without the drug treatment in Colon 38 (positive control) and OV2944-HM-1 tumor tissues on Day 1. (B) CD8 immunostaining at baseline without the drug treatment in Colon 38 (positive control) and HM-1 tumor tissues on Day 1. Scale bar, 100 µm. HM-1, OV2944-HM-1; IHC, immunohistochemistry; PD-L1, programmed death-ligand 1.

First, the HM-1 tumor was characterized at baseline using immunohistochemistry. HM-1 tumors exhibited low PD-L1 expression, while Colon 38 tumors were PD-L1-positive (Fig. 1A). CD8^+^ T cells were hardly observed in HM-1 tumors, whereas their infiltration was prominent in Colon 38 tumors (Fig. 1B). This indicated that HM-1 tumors exhibited PD-L1^low^ and immune desert-like phenotypes.

### Anti-PD-L1 antibody combined with anti-VEGF antibody improves tumor control compared with anti-VEGF antibody alone in an anti-PD-L1 insensitive HM-1 tumor model

**Figure 2.**
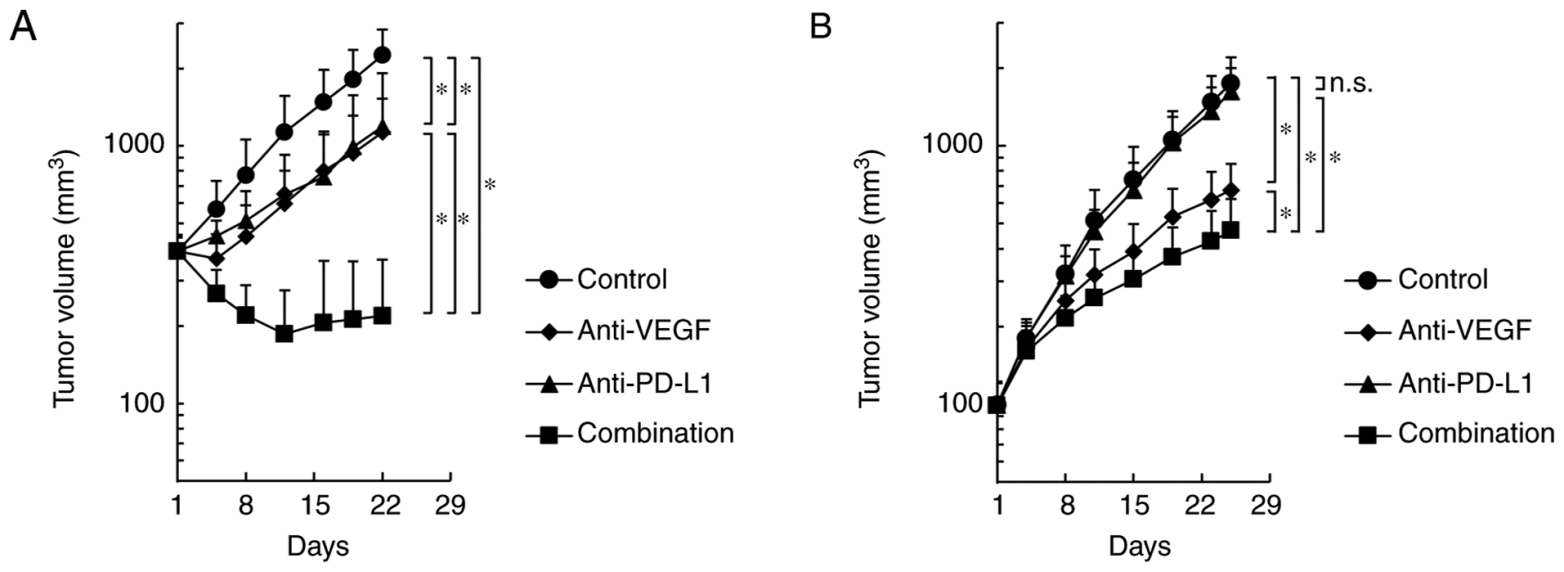
Antitumor activity of anti-PD-L1 antibody in combination with anti-VEGF antibody. Tumor growth curves. Mice bearing (A) anti-PD-L1-sensitive Colon 38 or (B) anti-PD-L1-insensitive OV2944-HM-1 tumors were randomly divided into four groups: Control, anti-VEGF, anti-PD-L1 and combination. Data are presented as the mean + SD. (A) n=8/group; (B) n=15/group. *P<0.05 (Wilcoxon rank sum test with Holm-Bonferroni correction). n.s., no significant difference; PD-L1, programmed death-ligand 1; VEGF, vascular endothelial growth factor.

In the Colon 38 model, both anti-PD-L1 antibody alone and anti-VEGF antibody alone significantly inhibited tumor growth compared with the control, and combination efficacy of anti-PD-L1 and anti-VEGF was shown (Fig. 2A). In the HM-1 model, the anti-VEGF antibody alone significantly inhibited tumor growth compared with the control; however, the anti-PD-L1 antibody alone did not significantly inhibit the tumor growth compared with the control (Fig. 2B). Notably, the anti-PD-L1 antibody, when combined with anti-VEGF, exhibited significantly stronger antitumor efficacy compared with anti-VEGF antibody alone, even in the anti-PD-L1 insensitive HM-1 model (Fig. 2B). Therefore, it was determined that the combination of anti-PD-L1 antibody plus anti-VEGF antibody exhibited more potent antitumor activity compared with single agent treatments in not only the anti-PD-L1 sensitive model, but also in the anti-PD-L1 insensitive model.

### Higher percentage of effector CD8^+^ T cells in the tumors treated with anti-PD-L1 antibody combined with anti-VEGF antibody in the HM-1 tumor model

**Figure 3.**
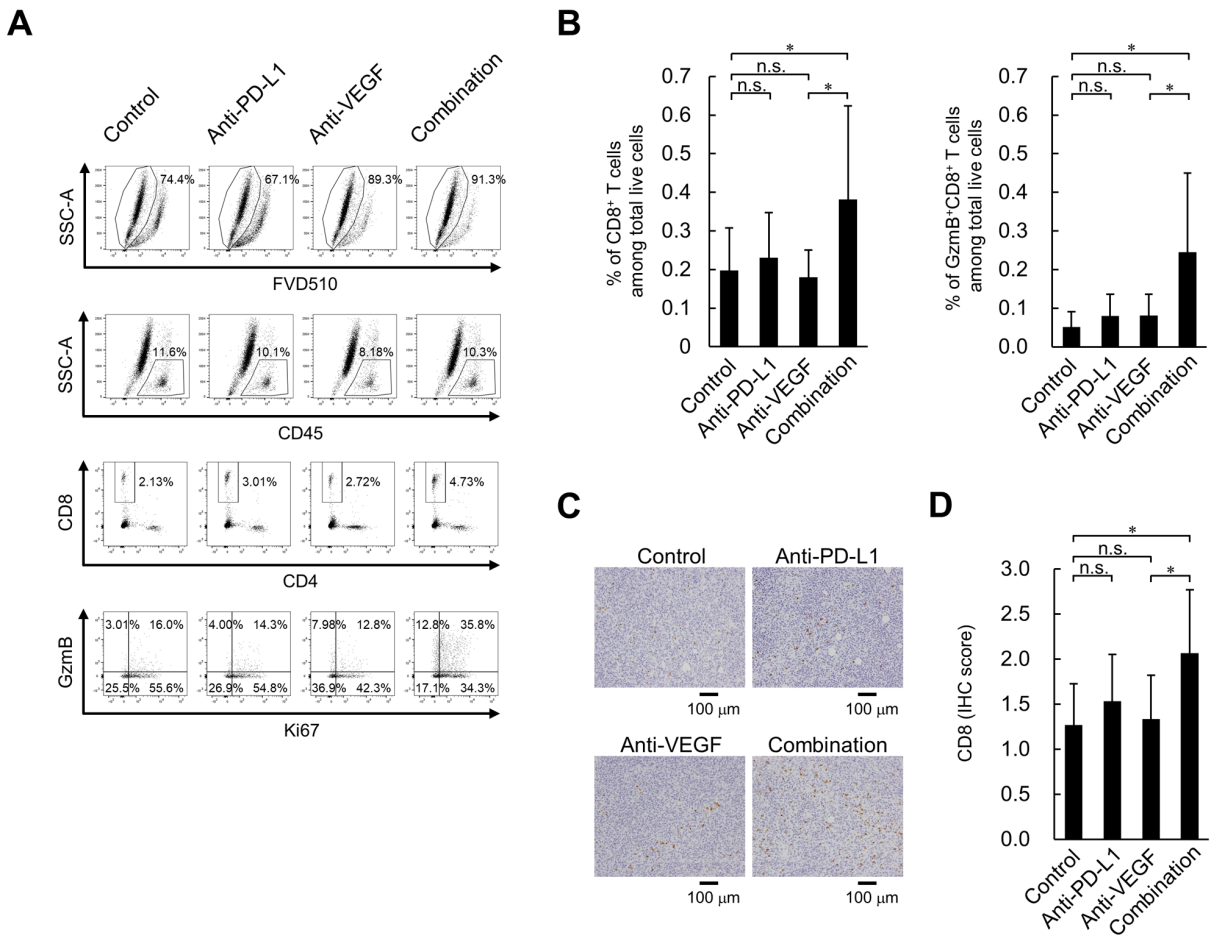
Effect of anti-PD-L1 antibody in combination with anti-VEGF antibody on percentage of tumor-infiltrating CD8^+^ T cells in the OV2944-HM-1 tumors. (A) Representative flow cytometric profiles of CD8^+^ T cells on Day 8. (B) Percentage of intratumoral CD8^+^ T cells and GzmB^+^CD8^+^ T cells on Day 8. These populations were determined using flow cytometry. Data are presented as the mean + SD. CD8^+^ T cells; control, n=15/group; anti-PD-L1, n=14/group; anti-VEGF, n=14/group; combination, n=15/group. GzmB^+^CD8^+^ T cells; n=15/group. (C) Tumor-infiltrating CD8^+^ T cells stained immunohistochemically with anti-CD8 antibody on Day 8. Scale bar, 100 µm. (D) Levels of tumor-infiltrating CD8^+^ T cells were indicated using IHC scores. Data are presented as the mean + SD. n=15/group. *P<0.05 (Wilcoxon rank sum test with Holm-Bonferroni correction). FVD510, fixable viability dye eFluor 510; GzmB, granzyme B; IHC, immunohistochemistry; n.s., no significant difference; PD-L1, programmed death-ligand 1; SSC-A, side scatter area; VEGF, vascular endothelial growth factor.

To investigate the effect of the anti-PD-L1 and anti-VEGF combination treatment on immune status, the present study analyzed the intratumoral status of CD8^+^ T cells on Day 8, when efficacy began to appear (Fig. 2B), using flow cytometry. No significant difference was observed in the intratumoral percentage of CD8^+^ T cells and GzmB^+^CD8^+^ T cells between the anti-PD-L1 antibody treatment and the control antibody treatment; however, there was a significantly higher percentage of CD8^+^ T cells and GzmB^+^CD8^+^ T cells in the anti-PD-L1 and anti-VEGF antibody combination treatment group compared with the group treated with anti-VEGF antibody alone (Fig. 3A and B). Additionally, immunohistochemical staining using an anti-CD8 antibody revealed that the combination of anti-PD-L1 and anti-VEGF antibody induced a significantly larger number of intratumoral CD8^+^ T cells compared with the anti-VEGF antibody alone (Fig. 3C and D). On Day 4, compared with the control treatment, anti-PD-L1 treatment both with and without anti-VEGF treatment resulted in higher percentages of CD8^+^ T cells and GzmB^+^CD8^+^ T cells in tumor tissues in the HM-1 model (Fig. S1).

**Figure 4.**
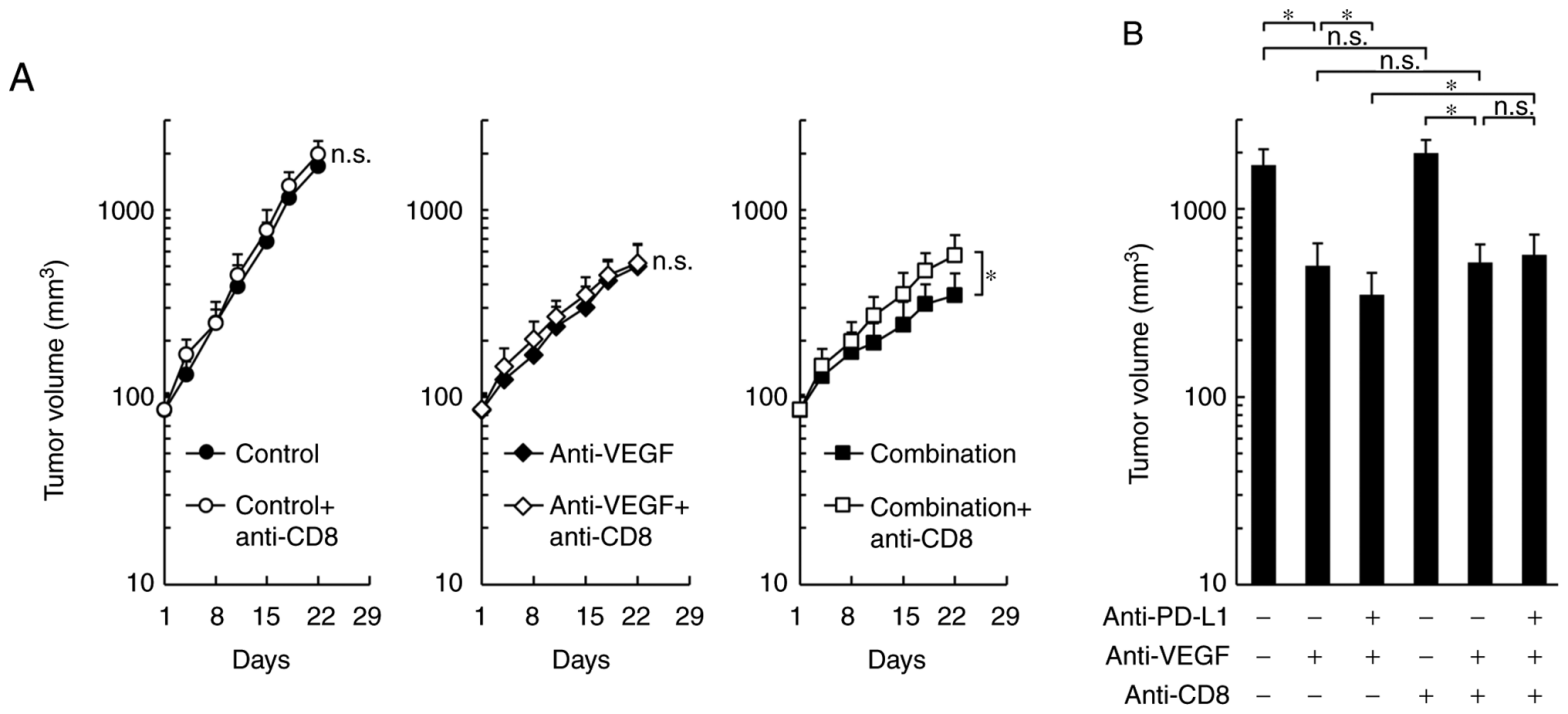
Effect of CD8 depletion on the antitumor effect of combination treatment with anti-PD-L1 antibody plus anti-VEGF antibody in OV2944-HM-1 tumors. (A) Tumor growth curves. Mice bearing HM-1 tumors were randomly divided into six groups: Control, control plus anti-CD8, anti-VEGF, anti-VEGF plus anti-CD8, combination and combination plus anti-CD8. (B) Volume of tumors treated with anti-PD-L1 mAb and anti-VEGF mAb plus anti-CD8 mAb on Day 22. Data are presented as the mean + SD (n=14/group). *P<0.05 (Wilcoxon rank sum test with Holm-Bonferroni correction). HM-1, OV2944-HM-1; mAb, monoclonal antibody; n.s., no significant difference; PD-L1, programmed death-ligand 1; VEGF, vascular endothelial growth factor.

By co-administration of anti-CD8 depleting antibody, the antitumor effects of the combination treatment were significantly reduced to the same level as that of the anti-VEGF antibody treatment alone, while control treatment and the anti-VEGF antibody treatment groups were not affected (Fig. 4A and B). These results suggested that the difference in the antitumor effect between combination treatment and anti-VEGF treatment may be caused by CD8^+^ T cells.

### Higher levels of intratumoral CXCR3 ligands in the combination treatment with anti-PD-L1 plus anti-VEGF group in the HM-1 tumor model

**Figure 5.**
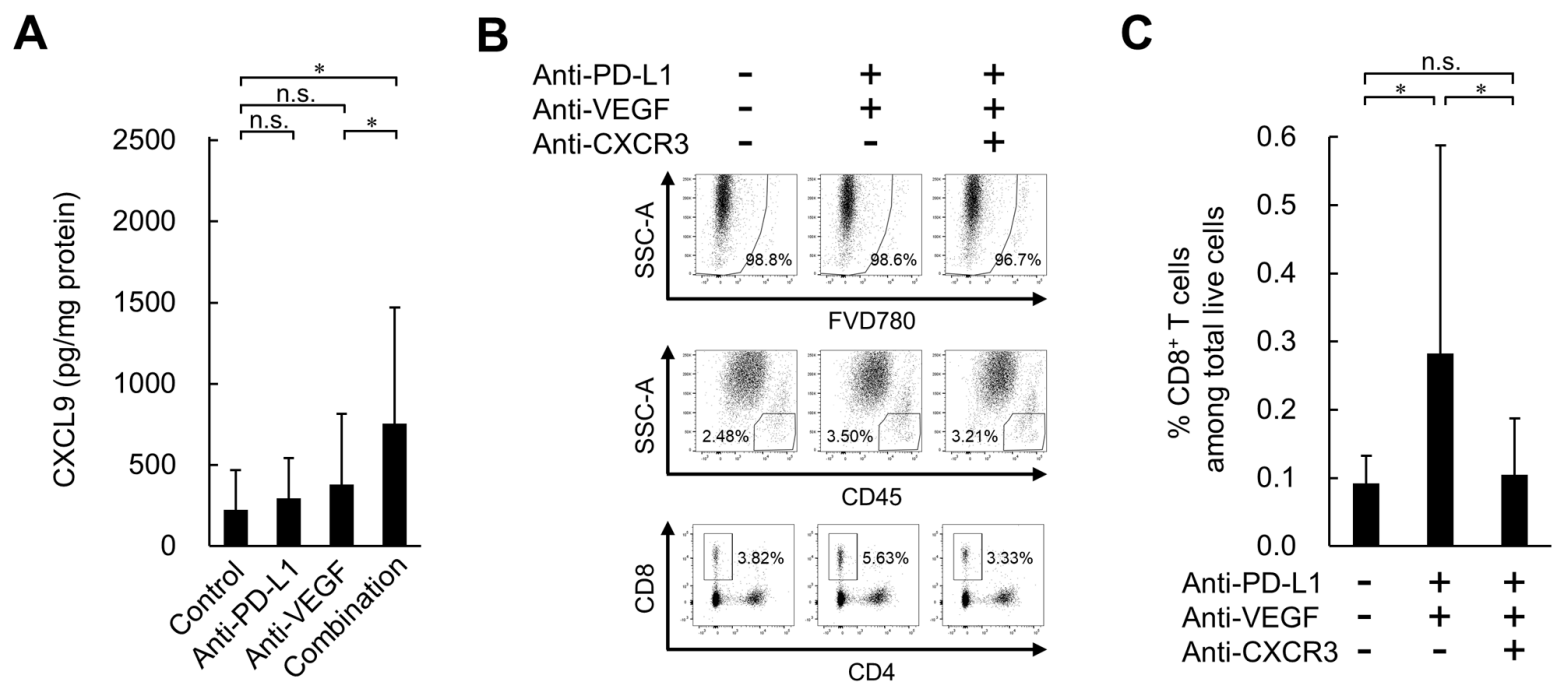
Effect of anti-PD-L1 antibody in combination with anti-VEGF antibody on expression levels of intratumoral CXCL9, and effect of CXCR3 blocking on tumor-infiltrating CD8^+^ T cells in the OV2944-HM-1 tumors. (A) Expression levels of intratumoral CXCL9 on Day 8 determined by ELISA. Data are presented as the mean + SD (control, n=15/group; anti-PD-L1, n=15/group; anti-VEGF, n=14/group; combination, n=15/group). (B) Representative flow cytometric profiles of CD8^+^ T cells on Day 8. (C) Percentage of intratumoral CD8^+^ T cells on Day 8. These populations were determined by flow cytometry. Data are presented as the mean + SD (n=16/group). *P<0.05 (Wilcoxon rank sum test with Holm-Bonferroni correction). CXCL9, C-X-C motif chemokine ligand 9; CXCR3, C-X-C motif chemokine receptor 3; FVD780, fixable viability dye eFluor 780; n.s., no significant difference; PD-L1, programmed death-ligand 1; SSC-A, side scatter area; VEGF, vascular endothelial growth factor.

To investigate the mechanism behind the increase in intratumoral CD8^+^ T cells caused by the combination of anti-PD-L1 and anti-VEGF, the present study focused on the CXCR3 ligands, CXCL9, CXCL10 and CXCL11, which have been reported to recruit CD8^+^ T cells into tumors (26–28). On Day 4, the protein levels of CXCL9 were significantly higher in the anti-PD-L1 antibody alone treatment group compared with the control treatment group (Fig. S2). Anti-PD-L1 treatment alone also exhibited a tendency to increase CXCL10, but this was not statistically significant. CXCL11 levels were not affected by any of the treatments. On Day 8, no significant difference was observed in the intratumoral protein levels of CXCL9 between the anti-PD-L1 antibody treatment and control treatment groups (Fig. 5A). On the other hand, significantly higher levels were observed in the anti-PD-L1 antibody treatment combined with the anti-VEGF antibody group compared with the anti-VEGF antibody treatment alone group. The combination treatment also exhibited a tendency to increase CXCL10, but it was not statistically significant (Fig. S2). The protein expression levels of intratumoral CXCL11 were not affected by any of the treatments. Since CXCR3 ligands have been reported to be induced by IFN-γ (28–30), the present study analyzed IFN-γ. On Day 4, significantly higher levels of intratumoral IFN-γ expression and percentages of IFN-γ^+^CD8^+^ T cells and IFN-γ^+^FoxP3^−^CD4^+^ T cells in tumor tissues were observed in the anti-PD-L1 antibody alone treatment group compared with control treatment group (Fig. S3). On Day 8, no significant difference was observed in these factors between the anti-PD-L1 antibody treatment and control treatment groups (Fig. S4). On the other hand, significantly higher levels of these factors were observed in the anti-PD-L1 antibody treatment combined with the anti-VEGF antibody group compared with the anti-VEGF antibody treatment alone group. When a blocking antibody for CXCR3 was co-administered, the number of intratumoral CD8^+^ T cells induced by the combination was reduced to the same level as that of the control (Fig. 5B and C). This result suggested that combination treatment promoted the trafficking of CD8^+^ T cells into tumors via the CXCR3 ligands, mainly CXCL9.

### Combination treatment with anti-PD-L1 plus anti-VEGF results in higher intratumoral MHC class I expression on tumor cells compared with anti-VEGF treatment alone in the HM-1 tumor model

**Figure 6.**
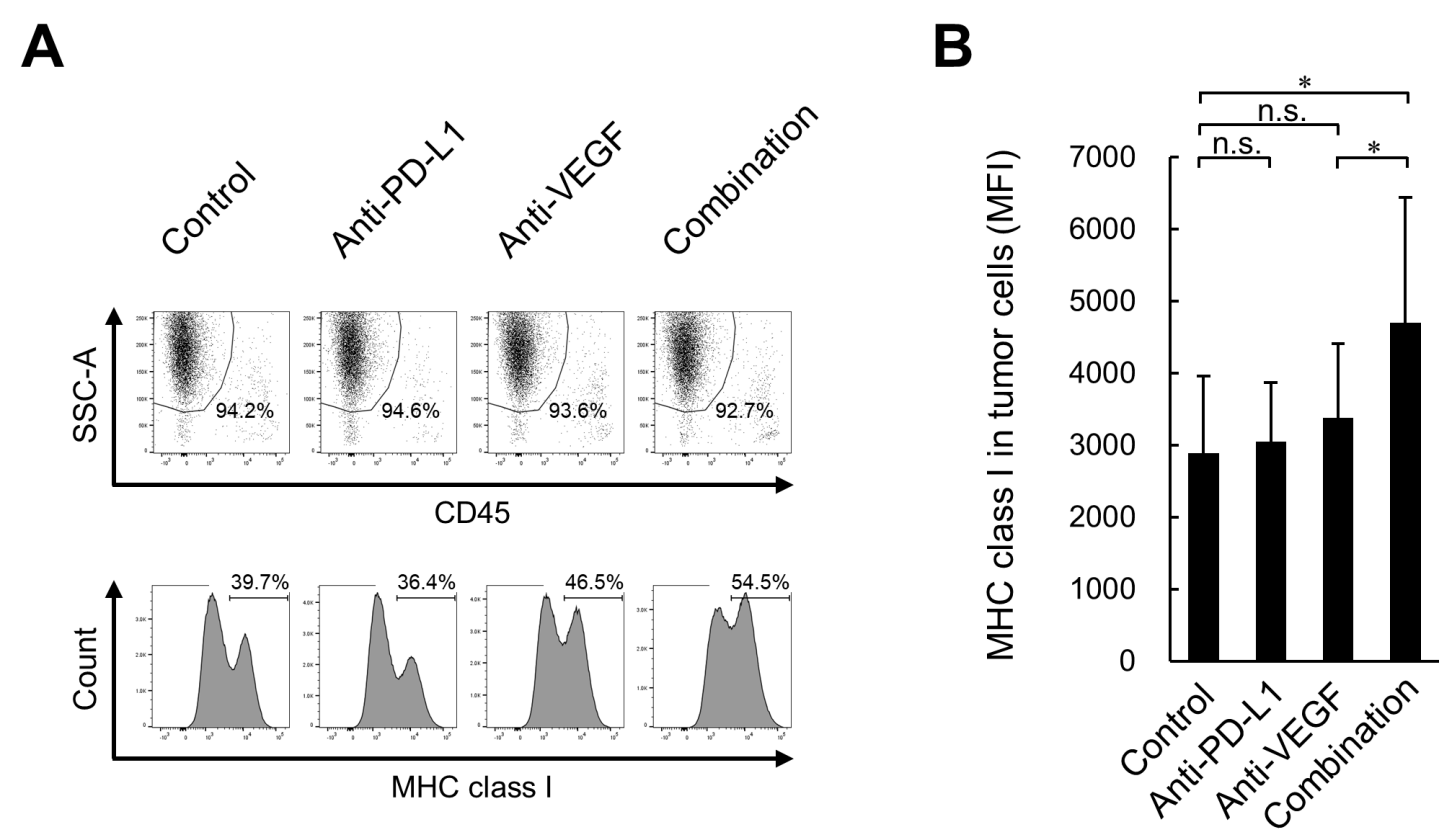
Effect of combination treatment with anti-PD-L1 antibody plus anti-VEGF antibody on the expression levels of MHC class I in the OV2944-HM-1 tumor cells. (A) Representative histograms of MHC class I on CD45^−^ cells on Day 8. (B) Expression levels of MHC class I on tumor cells on Day 8 determined by flow cytometry. Data are presented as the mean + SD (n=15/group). *P<0.05 (Wilcoxon rank sum test with Holm-Bonferroni correction). MFI, median fluorescence intensity; MHC, major histocompatibility complex; n.s., no significant difference; PD-L1, programmed death-ligand 1; SSC-A, side scatter area; VEGF, vascular endothelial growth factor.

Furthermore, to investigate whether treated tumor cells upregulate immune molecules implicated in antigen presentation to CD8^+^ T cells, the present study analyzed MHC class I (H-2K^k^) protein expression in tumors. Although no significant difference was observed in the expression levels of H-2K^k^ on tumor cells (CD45^−^, SSC^high^) between the anti-PD-L1 antibody treatment and the control treatment groups, significantly higher expression levels were observed in the anti-PD-L1 antibody treatment combined with anti-VEGF antibody group compared with the anti-VEGF antibody treatment alone group (Fig. 6A and B).

### Combination treatment with anti-PD-L1 plus anti-VEGF does not affect DC maturation, MDSC and Treg accumulation in tumors, or expression levels of ICAM-1, VCAM-1 and FasL in vascular endothelial cells in the HM-1 model

To investigate the mechanisms of the anti-PD-L1 and anti-VEGF combination treatment, the present study analyzed the following factors: DC maturation, MDSC and Treg accumulation in tumors, and expression levels of ICAM-1, VCAM-1 and FasL in vascular endothelial cells. In the HM-1 model, these factors were not affected by anti-PD-L1 or anti-VEGF either as monotreatment or in combination, except that a significantly higher ratio of CD8^+^ T cells to MDSCs was observed in the anti-PD-L1 antibody treatment combined with anti-VEGF antibody group compared with the anti-VEGF antibody treatment alone group (Figs. S5–S7).

### Anti-VEGF antibody suppresses MVD in the HM-1 model

The present study analyzed MVD on Day 8. No significant difference was observed in MVD in HM-1 tumor tissues on Day 8 between the anti-PD-L1 antibody treatment and the control treatment groups, and a significantly lower MVD was observed for the anti-VEGF antibody treatment both with and without anti-PD-L1 treatment compared with the control treatment. No significant difference was observed between the anti-VEGF antibody treatment and the combination treatment groups (Fig. S8).

## Discussion

Immune checkpoint inhibitors, such as anti-PD-L1/anti-PD-1, are a standard treatment for patients with several types of tumor; however, their remarkable efficacy may be limited to patients with PD-L1-positive and immune-inflamed (high CD8-positive rate) phenotypes (3,4). To overcome this limitation, numerous clinical trials have evaluated the efficacy of combinations with other agents, including anti-VEGF, in numerous types of cancer, such as non-small cell lung cancer, hepatocellular carcinoma and breast cancer (5,15,16). Among these combinations, that of atezolizumab (anti-PD-L1 antibody) and bevacizumab (anti-VEGF antibody) plus chemotherapy has been reported to have efficacy in the phase 3 IMpower150 trial for non-squamous non-small cell lung cancer (5). Although several possible combination mechanisms of anti-PD-L1/PD-1 and anti-VEGF have been reported using preclinical models, all of these models were anti-PD-L1 sensitive (17,18). Therefore, the present study examined the efficacy and mechanisms of the combination of anti-PD-L1 antibody and anti-VEGF antibody in the anti-PD-L1 insensitive HM-1 model. This model exhibited PD-L1^low^ and immune desert-like phenotypes, unlike the anti-PD-L1 sensitive Colon 38 model, which exhibited the PD-L1-positive and immune-inflamed like phenotypes. Notably, anti-VEGF antibody triggered the efficacy of anti-PD-L1 antibody in the anti-PD-L1 insensitive tumor model with PD-L1^low^ and immune desert-like phenotypes.

VEGF has been reported to exert immunosuppressive activity through the following mechanisms: Inhibition of DC maturation (9–12), accumulation of MDSCs in tumor (13), and decreases of ICAM-1 and VCAM-1 in vascular endothelial cells (31). VEGF blockade has been reported to promote antitumor immunity through the inhibition of Treg accumulation (14) and attenuation of tumor endothelial FasL expression (32). In the HM-1 model with the anti-PD-L1 insensitive phenotype, these phenomena were not observed. Additionally, VEGF has been investigated as a potential biomarker of responses to immune checkpoint inhibitor therapies (33). In particular, it has been reported in a pilot study that high pre-treatment serum VEGF levels in patients with advanced melanoma may predict poor responses to ipilimumab (anti-cytotoxic T-lymphocyte associated protein 4 antibody), while it was not identified as a predictor of poor responses in patients treated with pembrolizumab (anti-PD-1 antibody) alone or ipilimumab plus nivolumab (anti-PD-1 antibody) (33). Further prospective clinical studies with sufficient numbers of patients will be required to clarify the utility of VEGF as a predictor in these therapies.

On Day 8, the intratumoral higher percentages of CD8^+^ T cells and GzmB^+^CD8^+^ T cells were observed only when anti-PD-L1 was combined with anti-VEGF, and not without anti-VEGF. Since antitumor effects and MVD suppression were observed with an anti-VEGF single agent and these antitumor effects were not affected by CD8 depletion, it was suggested that the anti-VEGF single agent exhibits antitumor effects through anti-angiogenic activity in this model, not through CD8^+^ T cell-mediated immune enhancement. Since CD8 depletion reduced the antitumor effect of the combination treatment to the level of the anti-VEGF antibody treatment, it was considered that the difference between the antitumor effect of the anti-VEGF single agent and that of the combination treatment was due to the increase in intratumoral CD8^+^ T cells caused by the combination therapy.

The CXCR3 axis mainly positively regulates the infiltration of CD8^+^ T cells into tumors (26–28). In the HM-1 model, anti-PD-L1 treatment alone reportedly increases the percentage of activated CD8^+^ T cells in lymph nodes but not in tumors (34). This model showed ~10-fold lower levels of intratumoral CXCR3 ligands compared with the FM3A model, which also showed a PD-L1^low^ and immune desert-like phenotype but was sensitive to the anti-PD-L1 antibody (34). In a clinical study, the untreated tumors of patients who would later respond to atezolizumab exhibit elevated expression levels of IFN-γ and IFN-γ-inducible genes, including CXCL9 (35). Therefore, in the HM-1 model, it is possible that CD8^+^ T cells may not effectively infiltrate the tumor from the blood because of low intratumoral CXCR3 ligand expression, resulting in anti-PD-L1 insensitivity. In this model, on Day 8, intratumoral CXCL9 expression was upregulated by the anti-PD-L1 antibody combined with the anti-VEGF antibody, but not without the anti-VEGF antibody. This upregulation was associated with the higher percentage of intratumoral CD8^+^ T cells. CXCL10 exhibited the same tendency, but this was not statistically significant. The induction of an intratumoral percentage of CD8^+^ T cells by the combination treatment was reversed by CXCR3 blocking to the same level as control. These results suggested that, in the HM-1 model, the addition of the anti-VEGF antibody to the anti-PD-L1 antibody activated the CXCR3 axis, inducing CD8^+^ T cell infiltration into tumors. In addition to the CXCR3 axis, the CXCR5 axis has the potential to positively regulate the infiltration of CD8^+^ T cells into tumors (36,37). This is because CXCR5^+^CD8^+^ T cells have been reported to strongly infiltrate colorectal and pancreatic tumors and to exhibit strong cytotoxicity (36,37). In a future study, it would be interesting to further investigate the mechanistic role of this chemokine receptor and its ligands in the CD8^+^ T cell infiltration of tumors as induced by the combination of anti-PD-L1 antibody and anti-VEGF antibody.

IFN-γ is a pleiotropic molecule associated with anti-proliferative, pro-apoptotic and antitumor mechanisms (38). IFN-γ-induced intratumoral expression of CXCR3 ligands has been reported to alter the local distribution of T cells following immunotherapy (28–30). Both higher levels of IFN-γ expression and higher percentages of IFN-γ^+^CD8^+^ T cells and IFN-γ^+^FoxP3^−^CD4^+^ T cells in tumor tissue were observed in the combination of anti-PD-L1 antibody with anti-VEGF antibody group; this was similar to the expression levels of CXCR3 ligands and the percentages of CD8^+^ T cells. This suggested that IFN-γ in tumor tissues induced the expression levels of CXCR3 ligands, resulting in CD8^+^ T cell infiltration in the HM-1 model; however, the mechanism by which only the combination treatment was able to induce the higher levels of IFN-γ and CXCR3 ligands in this model requires further investigation. One possible explanation is that T cell re-activation by anti-PD-L1 under anti-VEGF-induced hypoxic conditions might contribute to increased IFN-γ-induced production of CXCR3 ligands, which is based on a report revealing that anti-VEGF induces hypoxia in tumors and that T cell activation under hypoxic conditions induces IFN-γ secretion (39,40).

On Day 4, for anti-PD-L1 both with and without anti-VEGF, higher percentages of CD8^+^ T cells and GzmB^+^CD8^+^ T cells were observed in tumor tissues in the HM-1 model. Notably, on Day 8, these higher percentages were only observed when the anti-PD-L1 antibody was combined with anti-VEGF antibody, but not without anti-VEGF antibody. This indicated that, in the HM-1 model, the intratumoral increase of activated effector T cells caused by the anti-PD-L1 single agent was transient, and was only maintainable in combination with the anti-VEGF antibody. A transient increase with anti-PD-L1 alone and a maintained increase with the anti-PD-L1 combined with anti-VEGF were also observed in the expression levels of CXCR3 ligands and IFN-γ, and in the percentages of IFN-γ^+^CD8^+^ T cells and IFN-γ^+^FoxP3^−^CD4^+^ T cells in tumor tissues. This may be why the anti-PD-L1 single agent failed to exhibit antitumor activity, and why anti-PD-L1 exhibited extra antitumor efficacy only when combined with anti-VEGF.

A number of cancer types acquire a phenotype of reduced expression levels of MHC class I molecules to escape recognition by cytotoxic T cells (41). In the present study, the combination treatment with anti-PD-L1 antibody plus anti-VEGF antibody upregulated the expression levels of MHC class I molecules on tumor cells, which is another possible mechanism of immune enhancement, and also maintained the accumulation of CD8^+^ T cells in tumor tissues. This was consistent with a report revealing that IFN-γ can upregulate MHC class I expression and enhance the cytotoxic T cell-mediated immune response (42), as well as with results revealing higher levels of IFN-γ expression for the combination treatment. The combination treatment may increase the presentation of tumor-antigens to specific cytotoxic CD8^+^ T cells.

In conclusion, to the best of our knowledge, this was the first study to reveal that the addition of anti-VEGF antibody overcomes anti-PD-L1 insensitivity in PD-L1^low^ and immune desert-like tumor models. This can be explained by the increase of intratumoral CXCR3 ligands leading to the increased infiltration of activated effector CD8^+^ T cells into tumor tissues. When combined with anti-VEGF antibody, therapy with anti-PD-L1 antibody is expected to exhibit more potent antitumor efficacy even in anti-PD-L1 insensitive patients with PD-L1^low^ and immune desert-tumors.

## Supplementary Material

Supporting Data

## Data Availability

The datasets used and/or analyzed during the current study are available from the corresponding author on reasonable request.
