# Supplementary material for: Anti-VEGF antibody triggers the effect of anti-PD-L1 antibody in PD-L1low and immune desert-like mouse tumors
Source: Oncol Rep. 2021 Dec 23;47(2):36. doi: 10.3892/or.2021.8247 (PMC8717122; doi:10.3892/or.2021.8247)

Figure S1. Effect of anti-PD-L1 antibody in combination with anti-VEGF antibody on the percentage of tumor-infiltrating CD8+ T cells on Day 4 in the OV2944-HM-1 tumors. Representative flow cytometric profiles of CD8+ T cells on Day 4 and percentage of intratumoral CD8+ T cells and GzmB+CD8+ T cells on Day 4. These populations were determined by flow cytometry. Data are presented as the mean + SD (n=15/group). *P<0.05 (Wilcoxon rank sum test with Holm-Bonferroni correction). FVD510, fixable viability dye eFluor 510; GzmB, granzyme B; n.s., no significant difference; PD-L1, programmed death-ligand 1; SSC-A, side scatter area; VEGF, vascular endothelial growth factor.

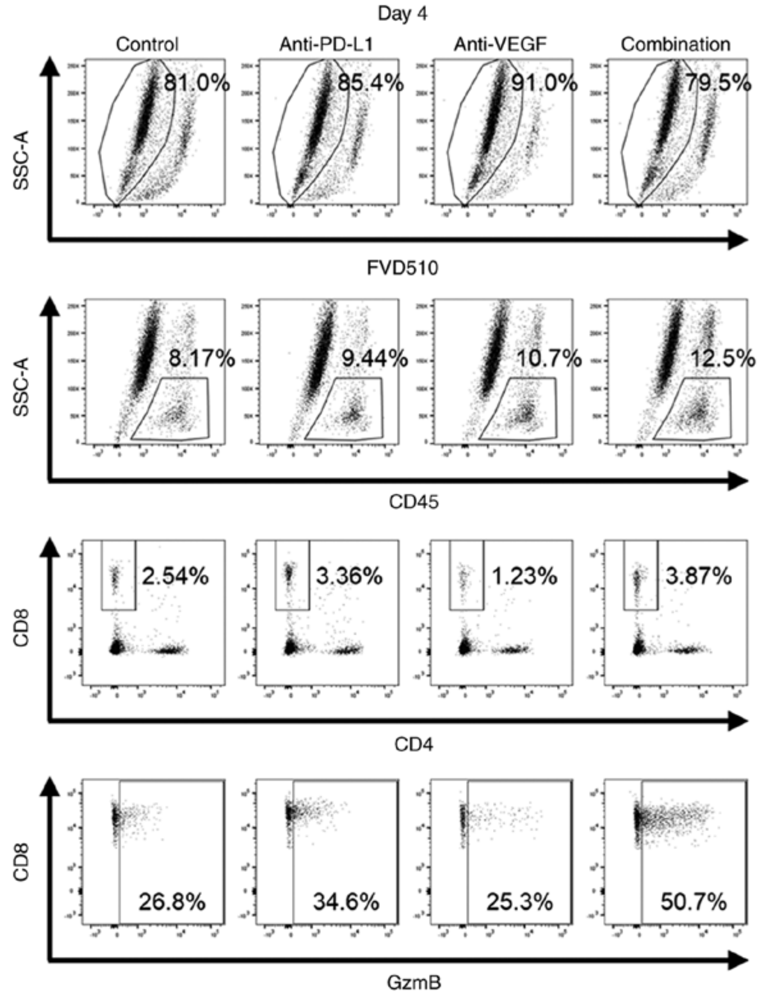

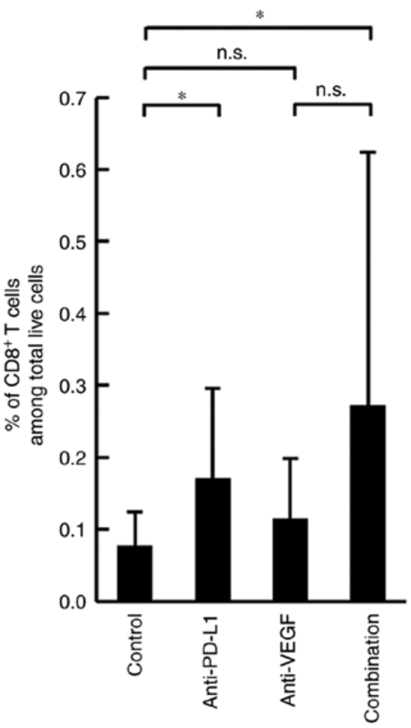

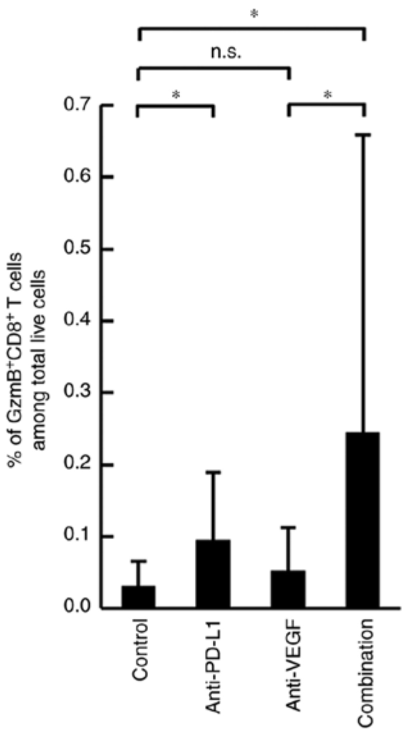

Figure S2. Effect of anti-PD-L1 antibody in combination with anti-VEGF antibody on the expression levels of intratumoral CXCL9, CXCL10 and CXCL11 in the OV2944-HM-1 tumors. Expression levels of intratumoral CXCL9, CXCL10 and CXCL11 on Days 4 and 8 were determined by ELISA. Data are presented as the mean + SD. Day 4; n=15/group. Day 8; control, n=15/group; anti-PD-L1, n=15/group; anti-VEGF, n=14/group; combination, n=15/group. *P<0.05 (Wilcoxon rank sum test with Holm-Bonferroni correction). CXCL, C-X-C motif chemokine ligand; n.s., no significant difference; PD-L1, programmed death-ligand 1; VEGF, vascular endothelial growth factor.

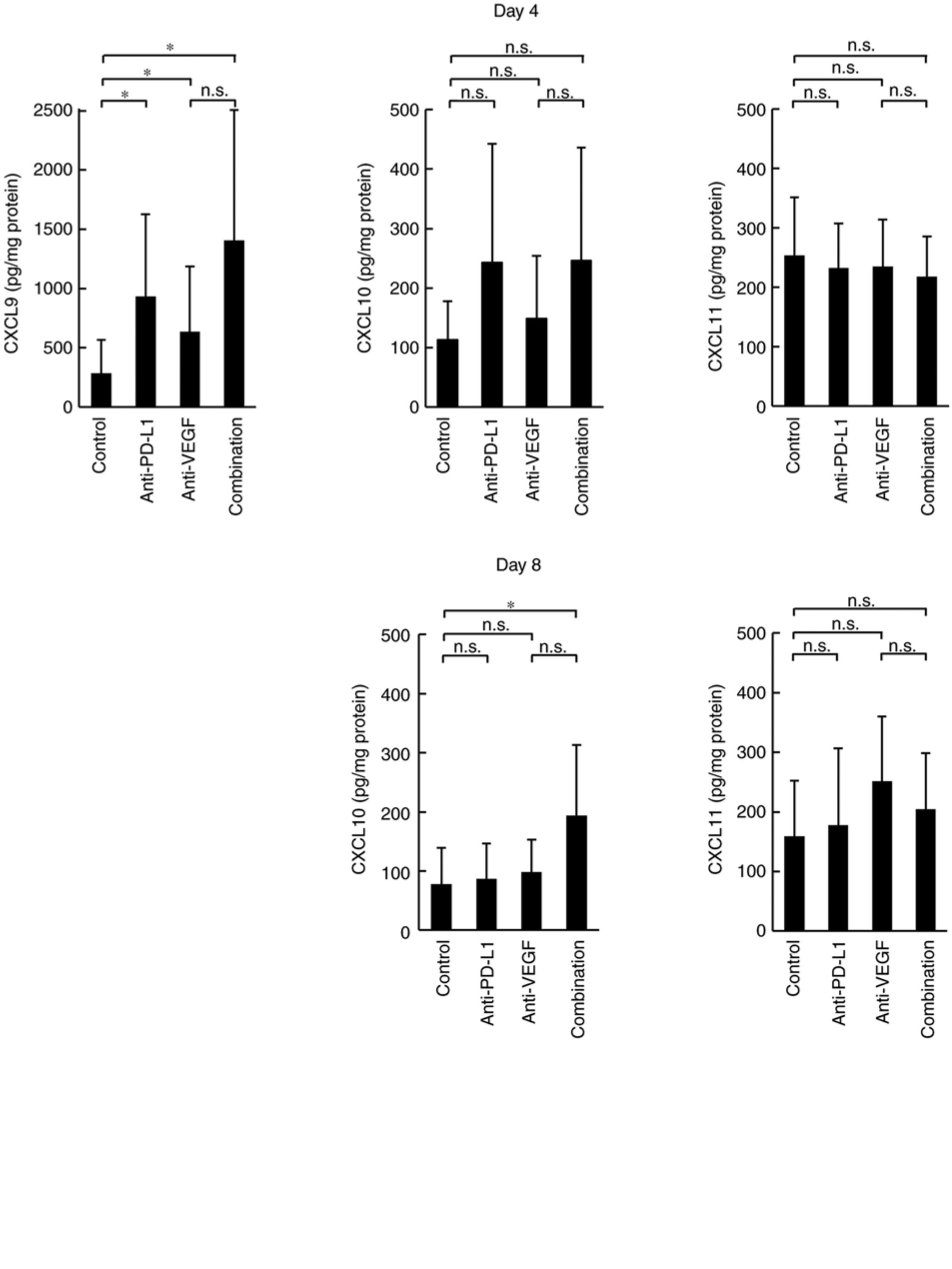

Figure S3. Effect of anti-PD-L1 antibody in combination with anti-VEGF antibody on IFN-γ expression on Day 4 in the OV2944-HM-1 tumors. (A) Expression levels of intratumoral IFN-γ on Day 4 were determined by ELISA. (B) Representative flow cytometric profiles of $CD8^+$ T cells and $FoxP3^-CD4^+$ T cells on Day 4, and percentages of intratumoral IFN-γ^+CD8^+ T cells and IFN-$\gamma^+FoxP3^-CD4^+$ T cells on Day 4. These populations were determined by flow cytometry. Data are presented as the mean + SD (n=15/group). $^*P<0.05$ (Wilcoxon rank sum test with Holm-Bonferroni correction). Foxp3, forkhead box P3; FVD510, fixable viability dye eFluor 510; n.s., no significant difference; PD-L1, programmed death-ligand 1; SSC-A, side scatter area; VEGF, vascular endothelial growth factor.

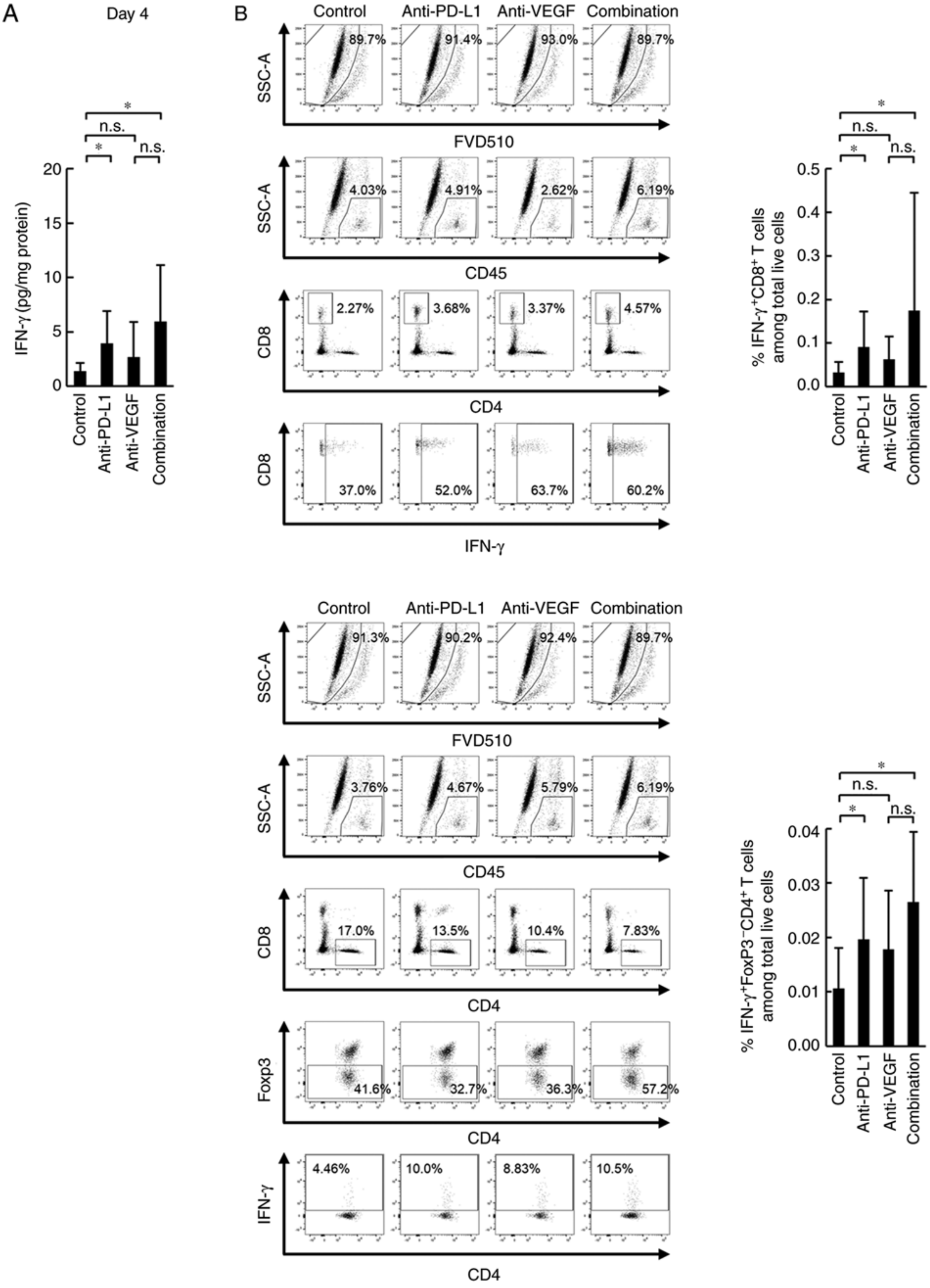

Figure S4. Effect of anti-PD-L1 antibody in combination with anti-VEGF antibody on IFN-γ expression on Day 8 in the OV2944-HM-1 tumors. (A) Expression levels of intratumoral IFN-γ on Day 8 were determined by ELISA. Data are presented as the mean + SD (control, n=15/group; anti-PD-L1, n=15/group; anti-VEGF, n=14/group; combination, n=15/group). (B) Representative flow cytometric profiles of $CD8^+$ T cells and $FoxP3^-CD4^+$ T cells on Day 8, and percentages of intratumoral IFN-γ^+CD8^+ T cells and IFN-$\gamma^+FoxP3^-CD4^+$ T cells on Day 8. These populations were determined by flow cytometry. Data are presented as the mean + SD (n=15/group). $^*P<0.05$ (Wilcoxon rank sum test with Holm-Bonferroni correction). Foxp3, forkhead box P3; FVD510, fixable viability dye eFluor 510; n.s., no significant difference; PD-L1, programmed death-ligand 1; SSC-A, side scatter area; VEGF, vascular endothelial growth factor.

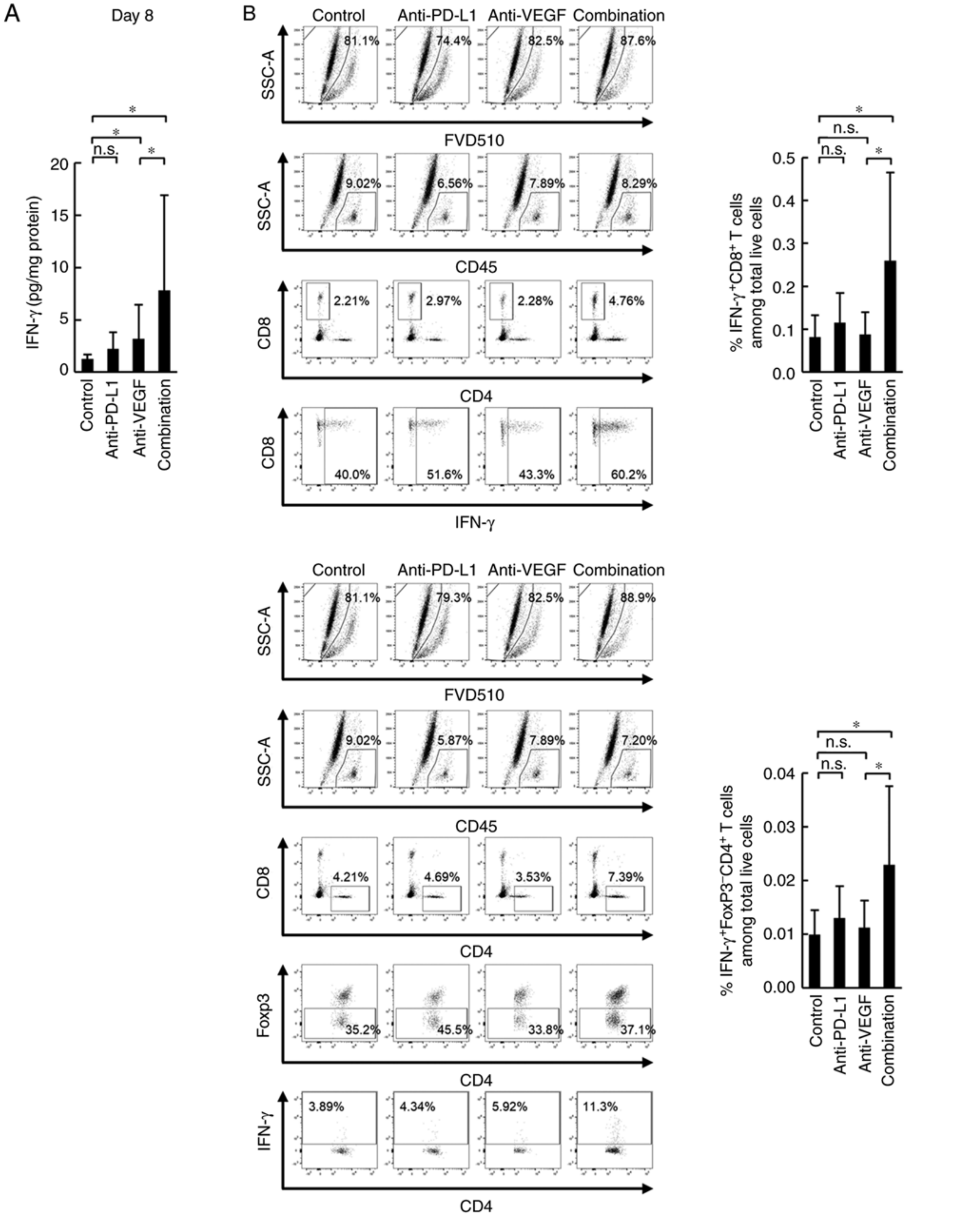

Figure S5. Effect of anti-PD-L1 antibody in combination with anti-VEGF antibody on percentage of mature dendritic cells in the OV2944-HM-1 tumors. Representative histograms of CD80 and CD86 on $CD11c^{+}$MHC class II^{+} cells (dendritic cells) on Day 8, and percentages of intratumoral $CD80^{+}CD11c^{+}$MHC class II^{+} cells and $CD86^{+}CD11c^{+}$MHC class II^{+} cells on Day 8. These populations were determined by flow cytometry. Data are presented as the mean + SD (n=15/group). Data were analyzed using the Wilcoxon rank sum test with Holm-Bonferroni correction. FVD780, fixable viability dye eFluor 780; MHC, major histocompatibility complex; n.s., no significant difference; PD-L1, programmed death-ligand 1; SSC-A, side scatter area; VEGF, vascular endothelial growth factor.

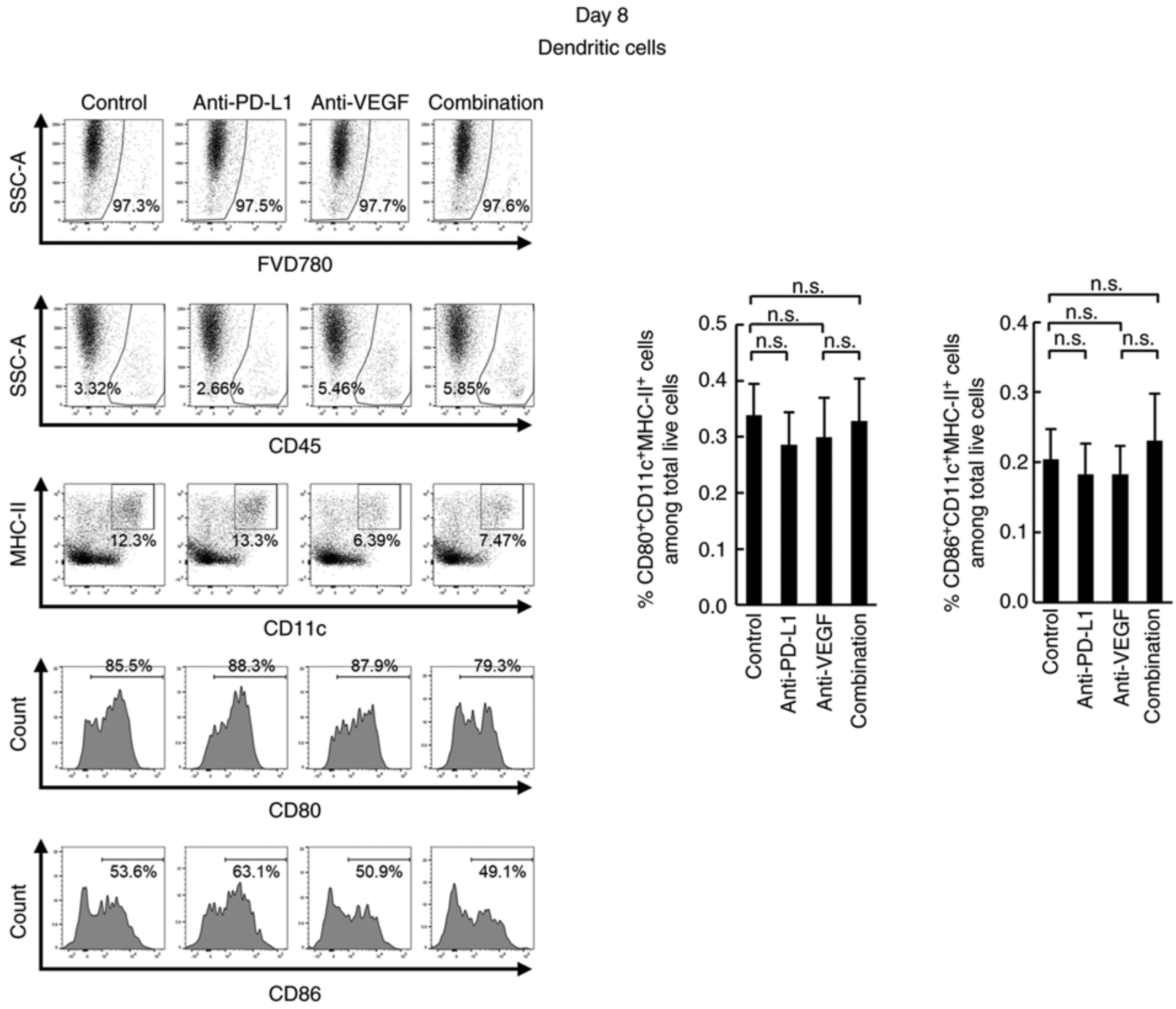

Figure S6. Effect of anti-PD-L1 antibody in combination with anti-VEGF antibody on Tregs and MDSCs in the OV2944-HM-1 tumors. Representative flow cytometric profiles, percentages of FoxP3^{+}CD4^{+} cells (Tregs) and CD11b^{+}Gr1^{+} cells (MDSCs), and ratio of CD8^{+} T cells to Tregs and MDSCs in tumors on Day 8. These were determined by flow cytometry. Data are presented as the mean + SD. Percentages of Tregs and MDSCs; n=15/group. Ratio of CD8^{+} T cells to Tregs and MDSCs; control, n=15/group; anti-PD-L1, n=14/group; anti-VEGF, n=14/group; combination, n=15/group. $^{*}P<0.05$ (Wilcoxon rank sum test with Holm-Bonferroni correction). Foxp3, forkhead box P3; FVD, fixable viability dye eFluor; Gr-1, granulocyte-differentiation antigen; MDSCs, myeloid-derived suppressor cells; n.s., no significant difference; PD-L1, programmed death-ligand 1; SSC-A, side scatter area; Tregs, regulatory T-cells; VEGF, vascular endothelial growth factor.

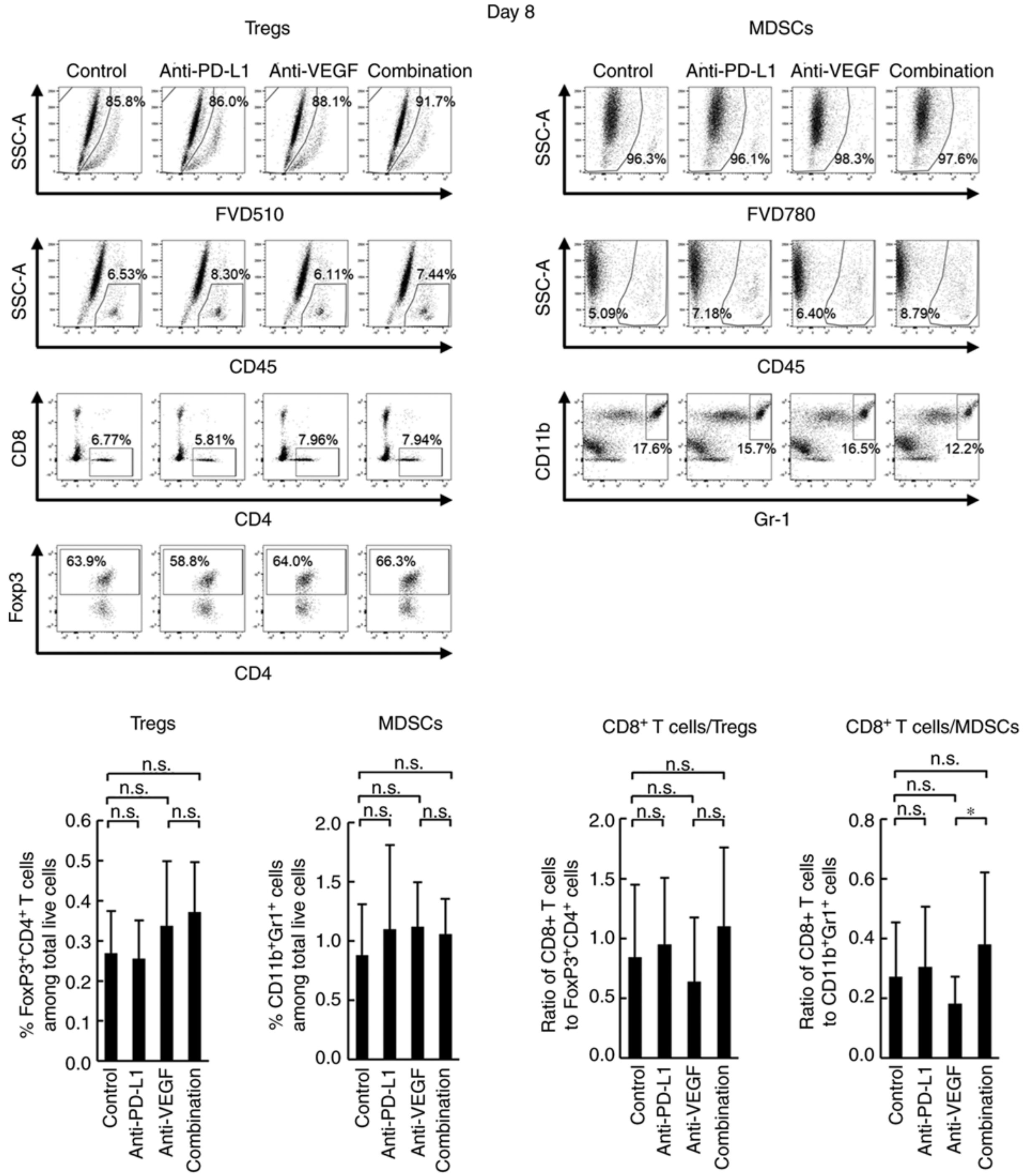

Figure S7. Effect of anti-PD-L1 antibody in combination with anti-VEGF antibody on expression levels of ICAM-1, VCAM-1 and FasL on tumor vascular endothelial cells in the OV2944-HM-1 tumors. Representative flow cytometric profiles of CD31$^+$ cells, and expression levels of ICAM-1, VCAM-1 and FasL on tumor vascular endothelial cells on Day 8 determined by flow cytometry. Data are presented as the mean + SD (n=6/group). Data were analyzed using the Wilcoxon rank sum test with Holm-Bonferroni correction. ICAM-1, intercellular adhesion molecule-1; MFI, median fluorescence intensity; n.s., no significant difference; PD-L1, programmed death-ligand 1; VCAM-1, vascular cell adhesion molecule 1; VEGF, vascular endothelial growth factor.

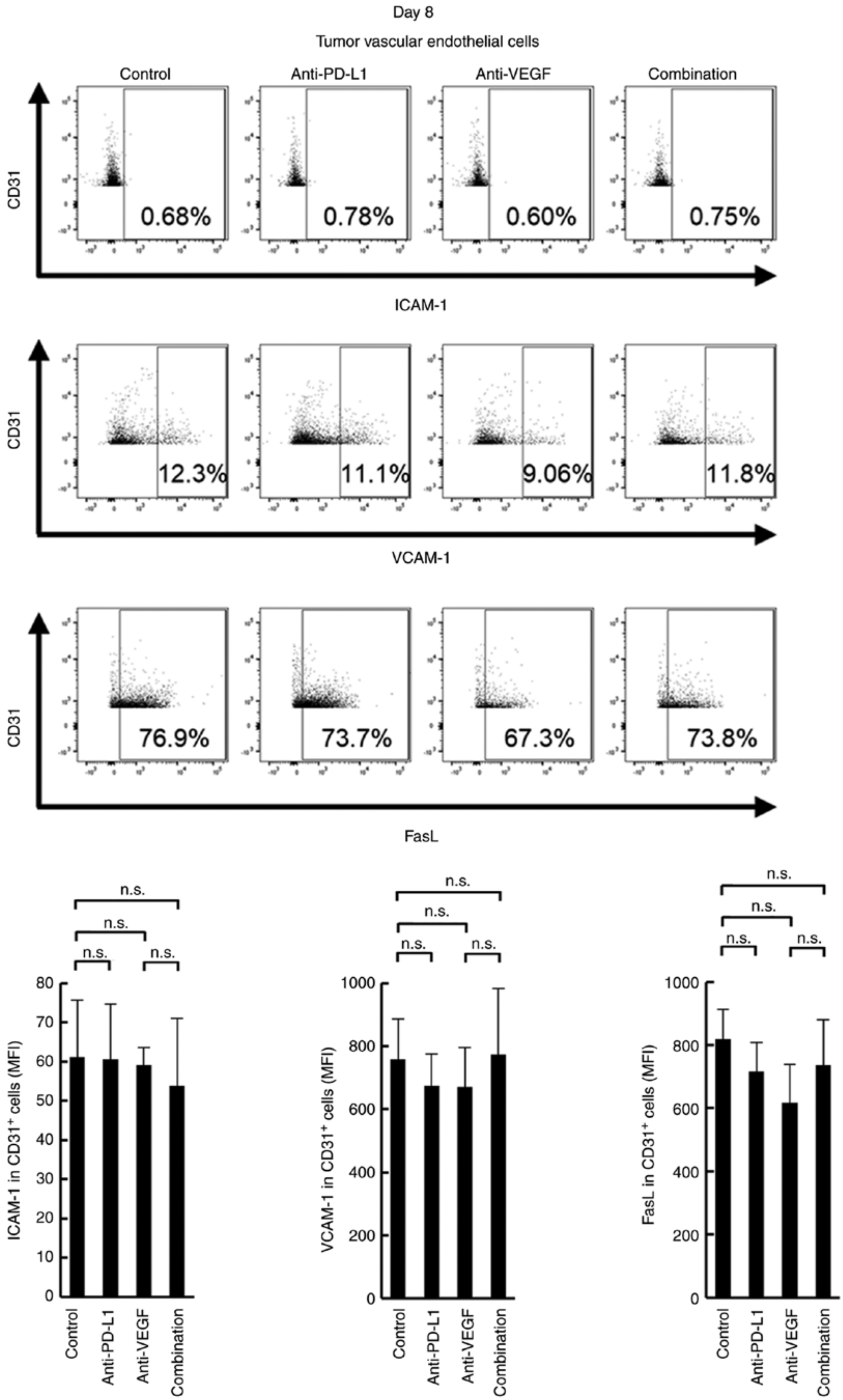

Figure S8. Effect of MVD in the OV2944-HM-1 tumors. (A) Tumor microvessels stained immunohistochemically with anti-CD31 in HM-1 tumor tissues on Day 8. Scale bar, 100 μm. (B) MVD in tumor tissue was determined by calculating the ratio of the CD31-positive area to the total observed area. Data are presented as the mean + SD (control, n=15/group; anti-PD-L1, n=15/group; anti-VEGF, n=15/group; combination, n=14/group). *P<0.05 (Wilcoxon rank sum test with Holm-Bonferroni correction). MVD, microvessel density; n.s., no significant difference; PD-L1, programmed death-ligand 1; VEGF, vascular endothelial growth factor.

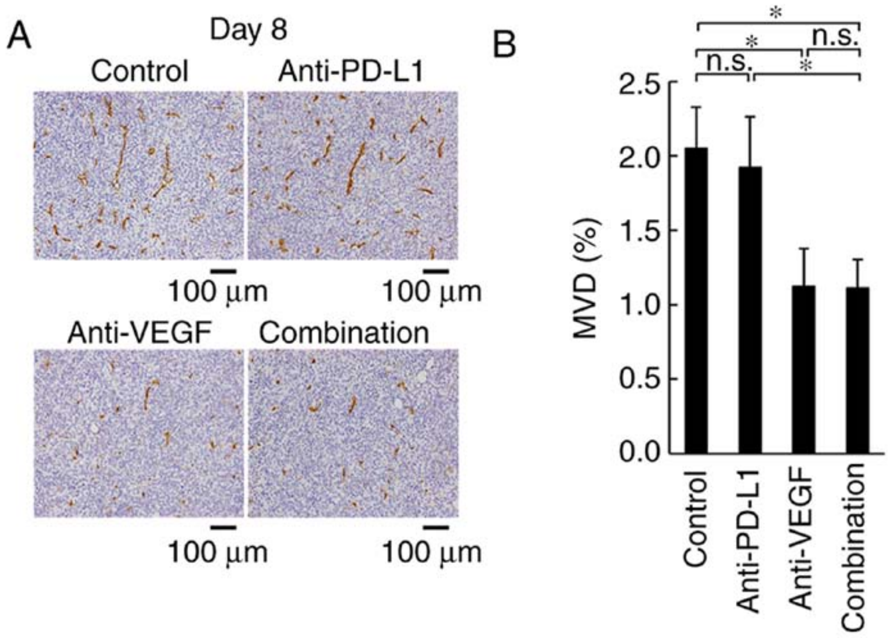

Supplement: Supporting Data [file Supplementary_Data.pdf]
